# Establishment of a method for *Lutzomyia longipalpis* sand fly egg microinjection: The first step towards potential novel control strategies for leishmaniasis

**DOI:** 10.12688/wellcomeopenres.14555.2

**Published:** 2018-08-15

**Authors:** Claire L. Jeffries, Matthew E. Rogers, Thomas Walker

**Affiliations:** 1 Department of Disease Control, Faculty of Infectious and Tropical Diseases, London School of Hygiene and Tropical Medicine, London, WC1E 7HT, UK

**Keywords:** Sand flies, leishmaniasis, egg microinjection, Wolbachia

## Abstract

Leishmaniases is a group of vector-borne parasitic diseases transmitted by sand flies that affects 1.3 million people across 98 countries, with limited control strategies due to the lack of an available vaccine and the emergence of insecticide resistance.  Novel control strategies that are being explored for mosquito-borne diseases, such as *Wolbachia *bacterial inhibition of pathogens and genetically modified insects (e.g. using CRISPR-Cas9 editing), rely on the ability to consistently inject eggs of the target species.  Here we present a novel method to obtain and inject preblastoderm sand fly eggs of the genus *Lutzomyia (Lu.)* *longipalpis*, the principle vector of zoonotic visceral leishmaniasis in South America. The procedures required to obtain sufficiently young *Lu. longipalpis *colony eggs are described alongside a microinjection technique that permits rapid injection and minimal handling of small sand fly eggs post-injection. Using a strain of *Wolbachia *as a ‘marker’ for successful injection, our protocol produced early generation *Wolbachia *transinfected *Lu. longipalpis *lines, demonstrating its potential as the first step for use in novel applied strategies for sand fly control.

## Introduction

Leishmaniases is a group of vector-borne tropical diseases transmitted by phlebotomine sand flies. The causative agent is a kinetoplastid protozoan from the genus *Leishmania,* which can cause a spectrum of diseases, collectively referred to as leishmaniasis. Clinical features range from simple, self-healing or large, chronic skin ulcers (cutaneous and mucocutaneous leishmaniasis) to potentially fatal infection of the liver and spleen (visceral leishmaniasis). The clinical symptoms exhibited are influenced by the species of the infecting parasites, the genetic background of the host and associated immunity, human migration and extrinsic factors such as reservoir animal hosts, human migration and control strategies ^1^. Leishmaniasis has been reported in 98 countries worldwide, putting an estimated 350 million people at risk of infection. Annually, leishmaniasis affects 1.3 million people, resulting in 20,000–40,000 deaths and an estimated 2.4 million Disability-Adjusted Life Years, where the highest burden on human health is amongst the poorest populations of society ^2^. Currently there is no human vaccine available and the choice of effective drugs is limited.

Globally, vector control represents the major arm for leishmaniasis elimination, mainly through indoor residual spraying (IRS). In South America, zoonotic visceral leishmaniasis, caused by *Leishmania* ( *Leishmania*) *infantum* (syn. *Leishmania chagasi*), is primarily transmitted by the neotropical sand fly *Lutzomyia (Lu.) longipalpis*. Although sand fly vector control strategies have historically been limited to small trials that have not reached large operational scale ^3^, recent trials have shown promise using a concentrated formulation containing the pyrethroid permethrin (an adulticide) and the larvicide pyriproxyfen. Although regular spraying can offer some protection to human populations ^4^, these programmes are often difficult to sustain, particularly in rural areas, where there are many potential resting sites requiring regular spraying. In Brazil, where over 90% of visceral leishmaniasis cases in South America occur, insecticide is applied only after a human case has been identified because of the logistics associated with spraying ^5^. Consequently, insecticide-treated bed nets (ITNs) or long-lasting insecticidal nets (LLINs) offer a suitable, cost-effective alternative to IRS. Deltamethrin-impregnated bednets were shown to reduce the human landing rates of *Lu. longipalpis* and the application of permethrin-impregnated netting (Olyset®) showed good efficacy in the first hour, however, the effectiveness diminished over time ^6^. A recent study using an adulticide-larvicide mixture of permethrin and pyriproxyfen (Dragon Max ^®^) in neighbouring Argentina was effective at significantly reducing the number of *Lu. longipalpis* ^7^. This formulation was effective for at least two weeks but further studies are required to determine if this formulation can have longer-term efficacy. However, the protection offered by treated nets in preventing human biting, and therefore *Leishmania* transmission, may be limited as *Lu. longipalpis* prefers to feed in the early part of the evening, before householders sleep under bed nets.

With the exception of *Phlebotomus argentipes*, the sand fly vector of anthroponotic visceral leishmaniasis in the Indian subcontinent ^4^, leishmaniasis vectors are highly susceptible to insecticides. However, the long-term feasibility of insecticide-treated materials is debatable due to logistical constraints (e.g., re-impregnation of materials), the potential for insecticide resistance ^8^ and the economic cost of these interventions ^9^. In addition, methods of environmental management to reduce wild reservoir host numbers, e.g. destruction of rodent burrows ^10^, have been limited. In endemic areas where dogs are domestic reservoirs of visceral leishmaniasis, insecticide-impregnated dog collars could be an effective and feasible strategy ^11^. The control of visceral leishmaniasis in the Americas has been further complicated by the urbanisation of *Lu. longipalpis* ^5^.

Research into novel non-insecticide based control strategies has been limited. The entomopathogenic fungus *Metarhizium anisopliae* was shown to have significant effects on egg hatching, survival of larvae and longevity of adult *Lu. longipalpis* ^12^. Attractive toxic sugar baits have shown efficacy against other leishmaniasis vectors, including *Phlebotomus papatasi* in Iran ^13^ and Morocco ^14^. Other potential control strategies that are yet to be explored include the use of the endosymbiotic bacterium *Wolbachia*, currently being used for mosquito biocontrol strategies given the ability of this naturally occurring bacterium to significantly reduce the vector competence of *Aedes (Ae.)* mosquitoes for arboviruses ^15– 20^. Alternative genetic strategies for mosquito control that could be applied to sand flies include the generation of sterile males that are then released to supress target populations ^21^ and the generation of transgenic lines that are refractory to pathogens using new genome editing tools such as CRISPR/Cas9 ^22^.

Mosquito embryo microinjection has played an integral role as the first step in the development of novel control strategies that are undergoing preliminary field trials in arbovirus endemic countries ( https://www.worldmosquitoprogram.org/, http://www.oxitec.com). *Wolbachia*-infected *Aedes* lines, including a superinfected line with two *Wolbachia* strains, have all been successfully generated using mosquito embryo microinjection ^15, 16, 23– 26^. Injection of young mosquito eggs has also been required for the successful genetic transformation of disease vectors ^27– 29^. The application of these novel vector control strategies for leishmaniasis requires the development of a protocol that would allow collection and injection of preblastoderm sand fly eggs. A key component of successful insect embryo injection is obtaining sufficient preblastoderm eggs that have not fully melanised as microinjection needles either are unable to penetrate or break upon contact with the hardened chorion of melanised eggs. Here we describe a method to obtain and microinject sand fly eggs of the genus *Lu. longipalpis*. We outline the steps required to collect sufficiently young *Lu. longipalpis* colony eggs and a method allowing rapid injection and minimal handling of small sand fly eggs post-injection. In order to determine the effectiveness of our protocol for targeting infection of the sand fly germline, we purified *w*Mel *Wolbachia* from *Drosophila melanogaster* eggs and used this endosymbiotic bacterium as a ‘marker’ for successful injection. Our protocol resulted in early generation *Wolbachia* transinfected *Lu. longipalpis* lines, demonstrating its potential to form the basis for novel control strategies for leishmaniasis sand fly vectors including both *Wolbachia*-based strategies and genetic modification.

## Methods

### *Lu. longipalpis* colony establishment and rearing

A laboratory strain of *Wolbachia*-negative *Lu. longipalpis* at the London School of Hygiene and Tropical Medicine was derived from a 30+ year closed colony, originating from Jacobina (Bahia state), Brazil. Sand flies were maintained at 26–28°C, 12:12 h light:dark cycle, ~80% relative humidity. Larvae were fed an equal part autoclaved mixture of ground-up laboratory rodent food pellets and rabbit faeces. Adult flies were given access to 25% (w/v) sucrose throughout their life and were fed on defibrinated rabbit or human blood to obtain eggs. Bloodfed female flies were encouraged to lay eggs in plaster of paris-lined oviposition pots for 6–7 days in total darkness. Following removal of adult fly bodies, eggs hatched over 3–4 days. The average life cycle duration from egg to egg was 5–6 weeks.

### Oviposition chambers for egg collection

**Figure 1.**
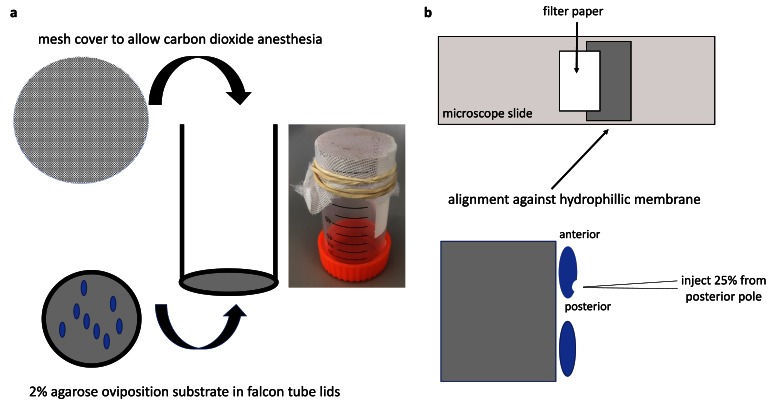
Schematic representation of the key steps in the egg microinjection protocol. ( **a**) *Lu. longipalpis* gravid females are removed from rearing cages using a mechanical aspirator and anaesthetised using carbon dioxide before placing on the side of an oviposition chamber. A mesh lid is secured and a falcon tube lid containing 2% agarose medium is screwed in to provide an oviposition substrate. After 45 minutes for oviposition, the mesh end of the chamber is placed on a carbon dioxide anaesthetising pad to allow exchange of oviposition substrate plates and removal of adult females prior to harvesting eggs from oviposition plates. ( **b**) *Lu. longipalpis* young eggs are aligned against a hydrophilic membrane in contact with moist filter paper to prevent eggs from desiccating. Eggs are injected with microcapillary needles ~25% of the length from the posterior pole and slides containing injected eggs are transferred to humidified chambers.

Gravid females from 3 days post-bloodfeed were removed from cages using a mechanical aspirator and anaesthetised using carbon dioxide by placing the aspirator chamber directly on a *Drosophila* Flystuff Ultimate Flypad. The flow of carbon dioxide was reduced relative to anaesthetising adult mosquitoes to ensure sand flies were not killed by the anaesthesia. An oviposition chamber was generated by removing the bottom of a 50mL falcon tube (Corning ^®^, CentriStar ^™^, Corning Inc.) and replacing this with mesh netting secured with an elastic band ( Figure 1a). A fine paintbrush was used to carefully transfer gravid anaesthetised females to the inside of an oviposition chamber laid on its side to avoid damage. Multiple oviposition substrates were made up in falcon tube lids allowing rapid change-over of substrate plates. During preparation, carefully pouring the substrate into the inner raised ring on the inside of the falcon tube lid, to form a substrate platform with a small gap around the edge, before allowing it to set, prior to use, enabled the falcon tube lids to be screwed easily and securely into the oviposition chambers.

As sand fly females typically lay their eggs in humid soil, rich in organic matter, and moisture can increase fecundity in laboratory colonies ^30^, we trialled three different substrates - including plaster of paris, the standard *Lu. longipalpis* colony larvae rearing substrate ^31^, and modified *Drosophila* embryo oviposition agarose-based substrates to determine if *Lu. longipalpis* would oviposit on either 2% apple juice agarose gel-based medium or 2% agarose gel prepared with water. For the plaster of paris substrate, a hole was punched through the falcon tube lid, prior to the plaster of paris being poured in to set, to enable humidity to be applied to the plates by placing them on wet paper towels. When appropriate, additional humidity was also applied to the plaster of paris lids through gently dropping small quantities of water on to the top of the plaster of paris, and allowing it to soak in at regular intervals to avoid it drying out.

Once transferred to the oviposition chambers, flies were left for 5 minutes or until there was evidence that they were actively walking or flying, before standing up the falcon tube on the lid. The chambers were then left for 45 minutes in the dark in a humidified box at 25°C to encourage oviposition. At the end of the oviposition period, sand flies were anaesthetised quickly using carbon dioxide for the shortest possible time and the oviposition substrate plates quickly exchanged to allow continued oviposition as required, and avoid mortality due to prolonged anaesthesia. Eggs were then harvested from the oviposition plates using very fine paintbrushes (Da Vinci Cosmotop-Spin, 10/0) to minimise damage and enable careful manipulation due to the small size of the eggs. The mortality of adult sand flies was recorded (dead flies were removed during oviposition plate exchanges) and the degree of embryo melanisation (light grey, medium grey, dark grey/black) was scored under a dissecting microscope. Selected females were maintained within oviposition chambers overnight by the addition of sugar soaked cotton wool to the mesh, and with replacement of oviposition substrate plates with empty falcon tube lids if it was desirable to prevent additional oviposition overnight. The flies were maintained between oviposition plate exchanges and overnight at 25°C within humidified incubators.

### Effects of larval rearing substrates on egg hatch rates

During the oviposition experiment, eggs collected on agarose oviposition plates from each group, at each time point, on days 3–7 post-bloodfeed were transferred, during egg counting and melanisation stage recording to either plaster of paris plates (2 replicates per time point as this is the standard larval rearing medium ^31^) or 2% water agarose gel plates (1 replicate per time point). The plates used for larval rearing substrate were prepared in the same way as the oviposition plates, (i.e. with substrate placed in falcon tube lids), and then each plate was screwed into complete falcon tubes, with humidity applied to the plaster of paris plates prior to use and maintained with damp paper towel placed on the bottom of the plates to prevent the plaster drying out. All hatching tubes were then placed in a falcon tube rack on its side and covered with a plastic bag within an incubator at 25˚C, with tubes regularly inspected to avoid insufficient or excess humidity.

### *Wolbachia* purification and egg injection

The *w*Mel strain of *Wolbachia* was purified from *Drosophila (D.) melanogaster* using modification of a method of *Wolbachia* purification described in Klasson *et al.* ^32^ by gently crushing x10 pairs of dissected ovaries using a plastic pestle in 100 μL of SPG buffer (218 mM sucrose, 3.8 mM KH _2_PO _4_, 7.2 mM K _2_HPO _4_, and 4.9 mM L-glutamate). Centrifugation of the homogenate at 500 x *g* removed cellular debris that would likely clog the microinjection needles. Purified *Wolbachia* in SPG buffer was kept on ice until injection, with subsequent DNA extraction and qPCR analysis performed on a sub-sample of the homogenate to confirm the presence of significant levels of *Wolbachia* bacteria. Embryonic microinjection was undertaken after alignment of young *Lu. longipalpis* eggs against a Hybond hydrophilic membrane as described in Walker *et al.* ^15^ and shown in Figure 1b. A very fine paintbrush (size 10/0) was required for alignment of eggs against the membrane. Hairs that fall off the adult sand flies during oviposition can stick to the eggs, making alignment and microinjection more difficult, and needle breakage more likely. Therefore, during alignment the paintbrush was kept wet and rinsed frequently in water to help adhere to the hairs and avoid them building up on the aligned eggs. Injection was carried out at x40 magnification under an Olympus IX73 microscope using an Eppendorf TransferMan ^®^ 4r micromanipulator, Eppendorf FemtoJet ^®^ 4x programmable microinjector and Eppendorf Femtotip II injection capillaries. After injection, microscope slides with eggs were immediately transferred to humidified boxes, prior to transfer of the eggs to dampened plaster of paris larval rearing medium.

### Isofemale line selection

Colony *Lu. longipalpis* females were screened for *Wolbachia* using universal *wsp* primers ^33^ prior to starting embryo injection experiments to confirm no evidence of natural resident *Wolbachia* strains. Isofemale lines were generated with modification of the colony rearing method. Emergent G0 females from microinjected eggs were housed with wild type colony males at a ratio of 10 males:1 female overnight to ensure insemination. The next day, G0 females were bloodfed and carefully transferred, individually, to oviposition chambers made from sterile polystyrene 7 mL bijou collection tubes (Costar) with a 1 cm thick moist plaster of paris base and netting top. Inside the tube a 1 cm x 2 cm strip of Whatman grade 4 filter paper was rested at a 45 degree angle to the plaster base to allow the fly to defecate their digested bloodmeal. A small cotton wool pellet soaked in sucrose solution was placed on top. When filled, the tubes were sealed inside a plastic box with moistened paper towel to maintain a high humidity and incubated in total darkness to encourage egg-laying. Sugarmeals were replaced every second day and excess moisture on the netting was blotted away. Following egg-laying, fly bodies and filter papers were removed and the emergent G1 larvae fed by depositing small amounts of larval food with sterile fine forceps next to the larvae.

Fly bodies were stored at –80°C until processing and DNA was extracted from G0 females that laid fertile egg batches using DNeasy Blood and Tissue Kits (QIAGEN) per manufacturer’s instructions. DNA extracts were eluted in a final volume of 100 μL and stored at –20°C. DNA extracts were screened using real-time PCR with primers specific for the *w*Mel strain of *Wolbachia* (forward primer: 5’-CAAATTGCTCTTGTCCTGTGG-3’, reverse primer: 5’-GGGTGTTAAGCAGAGTTACGG-3’) and with primers for a *Lu. longipalpis* VATPase gene (forward primer: 5’ - ACGTGACGAGCAAGCAGGGG, reverse primer 5’ – GCCGAGATCGTCCGACAGGC) to confirm successful DNA extraction. PCR reactions were prepared using 5 µL of FastStart SYBR Green Master mix (Roche Diagnostics), a final concentration of 1 µM of each primer, 1 µL of PCR grade water and 2 µL template DNA, to a final reaction volume of 10 µL. Prepared reactions were run on a Roche LightCycler® 96 System for 15 minutes at 95°C, followed by 50 cycles of 95°C for 15 seconds and 55°C for 30 seconds. Amplification was followed by a dissociation curve (95°C for 10 seconds, 65°C for 60 seconds and 97°C for 1 second) to ensure the correct target sequence was being amplified. PCR results were analysed using the LightCycler® 96 software (Roche Diagnostics). The female progeny from infected females were mated to uninfected colony males for 6 generations (G _0_–G _5_).

### Statistics

GraphPad Prism 7 was used to generate column bar graphs, Box and whisker plots and pie charts. Microsoft Excel for Mac (version 16.12) was used to generate adult survival curves.

## Results

### Oviposition substrate and embryo melanisation

**Figure 2.**
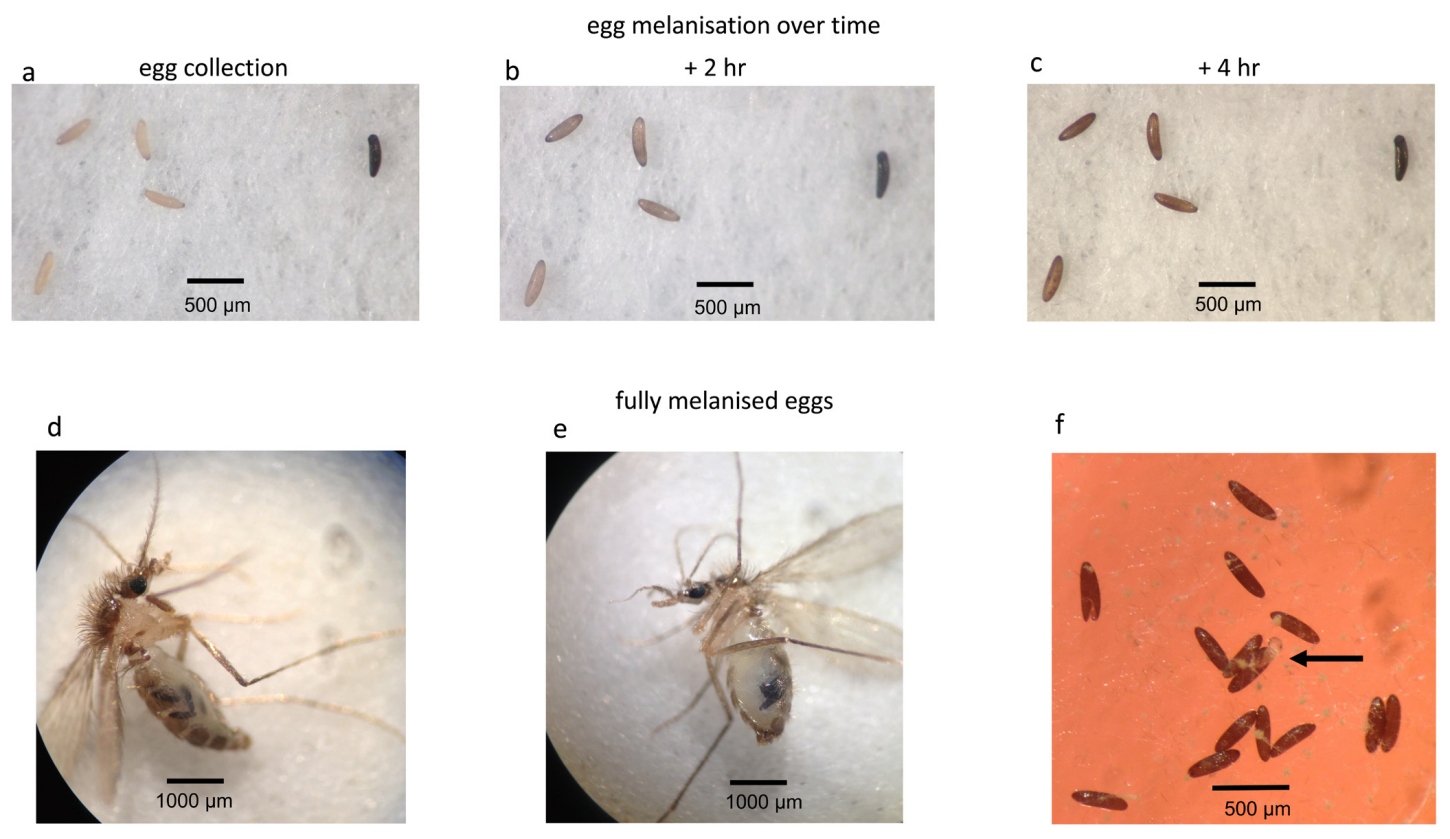
*Lu. longipalpis* egg melanisation over time. Different levels of melanised eggs were laid within oviposition chambers ranging from light grey (low melanisation) through to black eggs (fully melanised) ( **a**– **c**). Fully melanised eggs were also observed within the ovaries of gravid females ( **d**, **e**) and larvae were seen to hatch on oviposition substrates as denoted by the arrow ( **f**).

Preliminary tests were carried out to investigate the optimal methods to obtain large numbers of eggs suitable for microinjection. Initially three substrates - 2% apple juice agarose gel, 2% water agarose gel and moist plaster of paris - were compared, with oviposition chambers kept in humidified boxes, either in the light or placed in the dark. Trials included the addition of food colouring to the plaster of paris to better visualise un-melanised eggs (translucent to light grey in colour). Variations in the number of adult sand flies per oviposition chamber were also tested. Observations were made on the oviposited eggs and the survival of adult females. Like mosquitoes, *Lu. longipalpis* eggs melanised over a period of approximately 4 hours going from a translucent light colour to dark black ( Figure 2a–c). However, a significant proportion of fully melanised mature eggs (black in colour) laid within a 45-minute oviposition period were also observed. Fully melanised eggs were also present in the abdomens of gravid sand flies ( Figure 2d–e) highlighting that sand fly eggs can fully melanise prior to oviposition, and that at oviposition, there can be variability in the stage of melanisation, and therefore development, of eggs from the same female – an observation not seen in mosquitoes. Larvae hatching on oviposition substrates were also observed shortly after collection from females that laid fully melanised eggs ( Figure 2f), confirming that gravid sand flies can retain viable mature eggs until an appropriate substrate is available. These initial tests indicated that the optimal conditions for oviposition and adult longevity were obtained when using 2% water agarose gel as the oviposition substrate, with approximately 15 adult sand flies per oviposition chamber and when the flies were kept in the dark between oviposition plate exchanges. This combination of conditions was therefore used for further embryo collections.

### Duration of the egg collection period and timing of injectable egg collection

**Figure 3.**
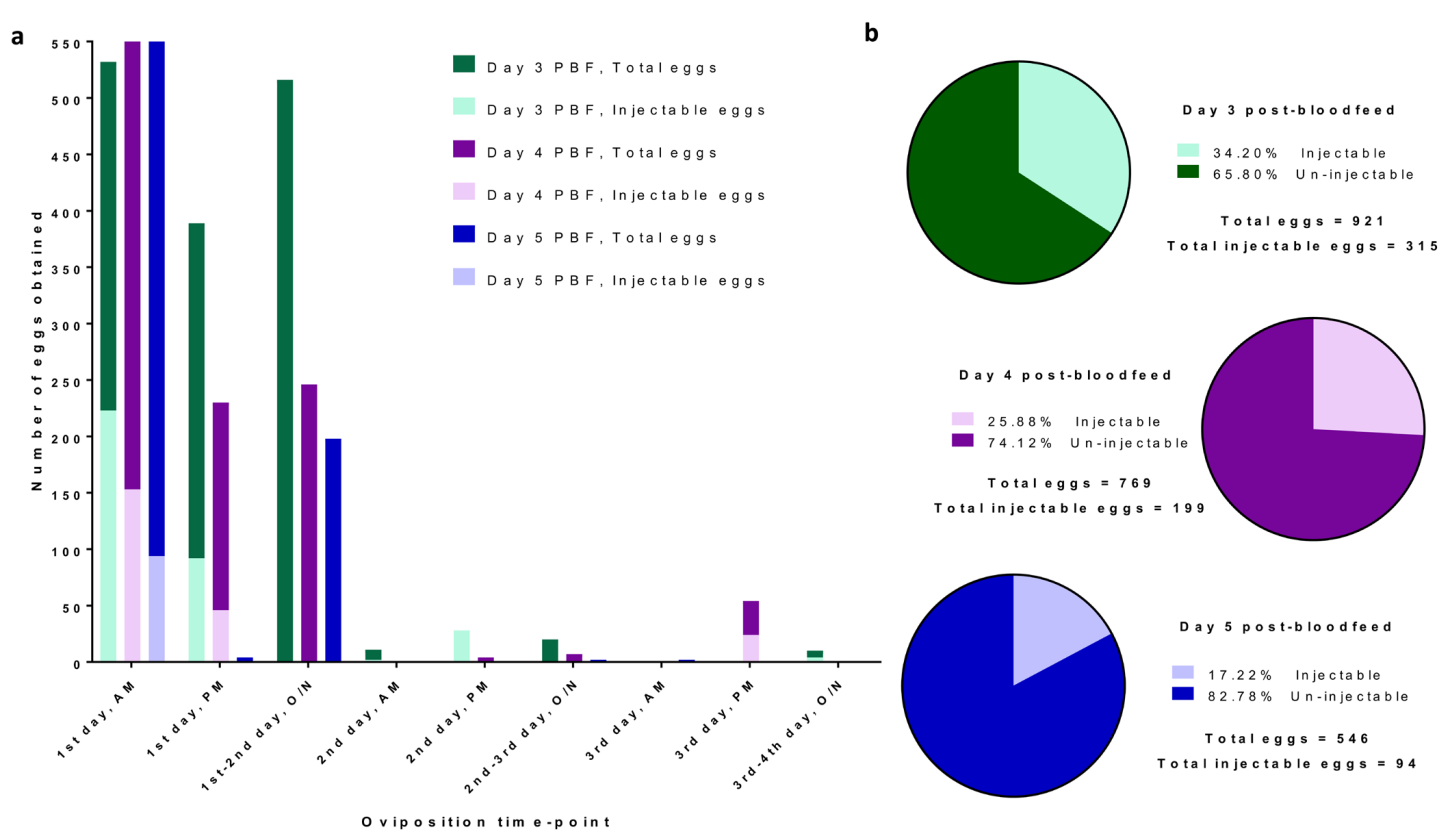
Timing of gravid *Lu. longipalpis* females oviposition and collection of injectable eggs. ( **a**) The total number of eggs oviposited, overlaid with the number of injectable eggs (light to medium grey in colour from melanisation) eggs obtained, per oviposition time point, per group setup for the first oviposition on either day 3, 4 or 5 post-bloodfeed (PBF). The total number of eggs from day 3 PBF = dark green, injectable eggs from day 3 PBF = light green, total eggs from day 4 PBF = purple, injectable eggs from day 4 PBF = pink, total eggs from day 5 PBF = dark blue, injectable eggs from day 5 PBF = light blue. ( **b**) The total numbers of eggs collected on the first oviposition day per group (first oviposition on day 3–5 post-bloodfeed comparing injectable eggs (light to medium grey in colour from melanisation) vs. uninjectable eggs (dark grey/black from melanisation). Day 3 PBF injectable eggs = light green (uninjectable dark green), day 4 PBF injectable eggs = pink (uninjectable purple), day 5 injectable eggs = light blue (uninjectable dark blue).

The temporal variation in the ability to obtain sufficient eggs to undertake microinjection experiments from one bloodfed cage of *Lu. longipalpis* (approximately 200 bloodfed females) was investigated. This involved setting up three replicate groups of females (15 females per chamber) on day 3 post-bloodfeed, 3 replicate groups for the first time on day 4, and 3 groups for the first time on day 5 post-bloodfeed. Each group was initially setup at 9am on the respective day of first oviposition, with agarose oviposition plate exchanges made at 1pm, 5pm and 9am the following morning, and continuing each day with these time intervals until all adult flies had died. At each plate exchange, both the total number of eggs collected per oviposition time period and the number of young eggs that would be suitable for injection (light to medium grey stage of melanisation) was recorded ( Figure 3a), as well as the survivorship of gravid *Lu. longipalpis* adult females during oviposition. The majority of injectable eggs was laid on the first exposure to oviposition substrate across all groups (223, 153 and 94 injectable eggs for Day 3, Day 4 and Day 5 groups, respectively) and the greatest proportion of injectable eggs obtained in a day was provided by those flies setup on day 3 post-bloodfeed ( Figure 3b). As fully melanised eggs cannot be used for microinjection, and the ability to obtain a large number of light to medium colour eggs within a day increases the efficiency of the injection process, collection of eggs on day 3 post-bloodfeed was considered optimal for both injection and survivability post-injection.

### Female survival after oviposition

**Figure 4.**
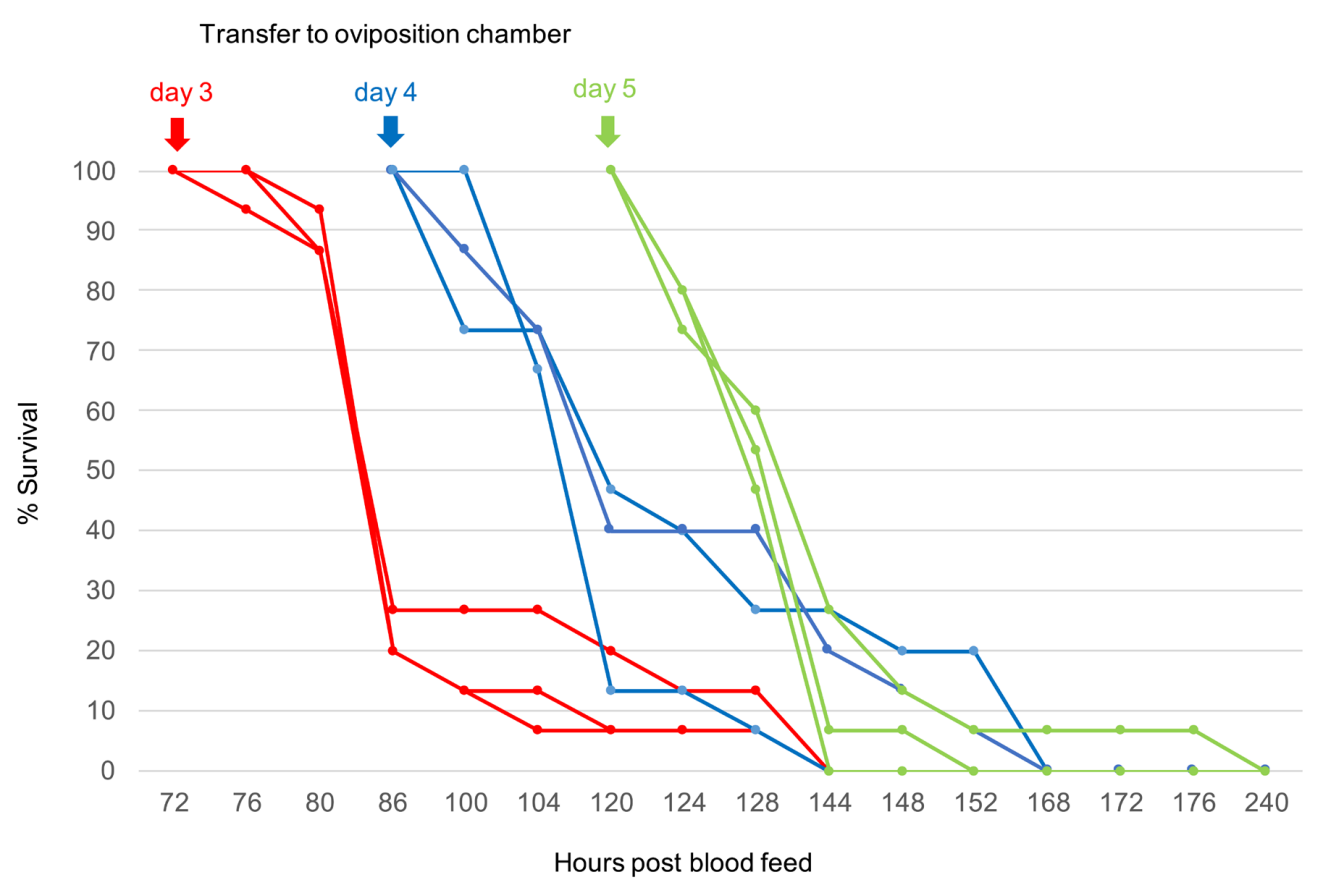
Survival rates of gravid *Lu. longipalpis* females during oviposition. 3 replicate groups of 15 flies were setup in oviposition chambers at day 3 (red), day 4 (blue) and day 5 (green) post-bloodfeed and subsequent mortality was recorded over time.

Egg microinjection experiments that either attempt to transinfect *Wolbachia* or to create transgenic lines require the successful generation of isofemale lines. This is dependent on females bloodfeeding and surviving (at the very least) long enough through a single gonotrophic cycle to oviposit the next generation of eggs. In mosquitoes, multiple gonotrophic cycles allows for the collection of progeny from older female mosquitoes, providing multiple chances and greater security that the next generation can be obtained, even if no eggs are produced from the first bloodfeed. To assess this for sand flies, the mortality of gravid females of varying ages was recorded (3–5 days post-bloodfeed) during egg collection. High rates of mortality were found regardless of the time post-bloodfeeding at which the flies were transferred to oviposition chambers. As shown in Figure 4, rapid mortality within 24 hours was observed for replicate groups of flies removed from colony cages and exposed to oviposition chambers. Although these survival results could suggest that significant mortality occurred from manipulation and exposure to oviposition chambers, high adult female mortality in colony *Lu. longipalpis* shortly after egg-laying was also observed.

### Hatch rates on larval rearing substrates

**Figure 5.**
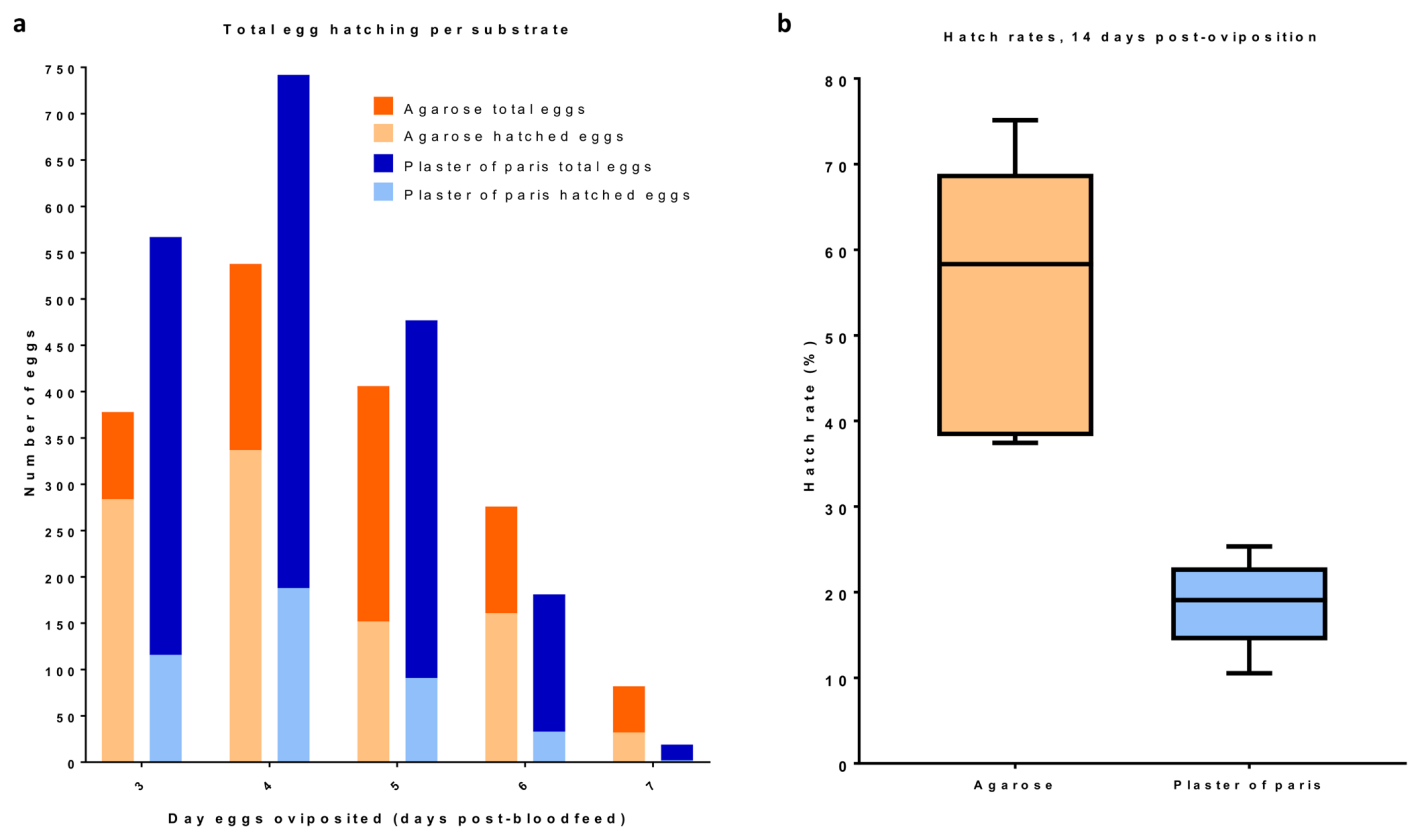
Hatch rates of *Lu. longipalpis* eggs transferred to either 2% agarose or plaster of paris as the larval rearing substrate. ( **a**) The total numbers of eggs, overlaid with the number of hatched eggs per substrate for each oviposition day. ( **b**) Box and whisker plot of hatch rates at 14 days post-oviposition across all days per substrate.

In order to optimise conditions for successful egg survival and larval hatching post-injection, the effect of larval rearing substrate on hatching was also investigated. Hatch rates were determined 14 days post-oviposition ( Figures 5a and 5b). An overall hatch rate of 57.5% of eggs maintained on agarose, across all oviposition days, compared to only 21.7% of eggs placed on plaster of paris, with a minimum of 37.4% for agarose and a maximum of 25.3% for plaster of paris demonstrates there is a clear improvement in hatch rates when agarose is used as the larval rearing medium over plaster of paris. This improvement may be as a result of the more constant humid environment provided by the agarose gel, providing an optimum environment for the eggs.

### Microinjection of *Wolbachia* purified from *D. melanogaster*

No evidence for natural *Wolbachia* strain infections was seen using PCR screening of the *Lu. longipalpis* colony prior to egg injection experiments. The *w*Mel strain of *Wolbachia* purified from the ovaries of *D. melanogaster* flies was then injected into the posterior poles of young *Lu. longipalpis* eggs. The injection volume and pressure was determined empirically during injections due to the variable physiology of *Lu. longipalpis* eggs. Slightly desiccated eggs, achieved by blotting of the hydrophilic membrane using filter paper, were re-inflated upon injection without significant flow of cytoplasm back up the injection needle. As *Lu. longipalpis* eggs are 300- 500 µm in length ^31^ (approximately 50% of the length of *Culex* or *Aedes* mosquito eggs) care was taken to identify the optimal location for injection. For infection of the germline, injection was carried out as near to the posterior pole as possible without significant damage. The posterior pole regions of eggs were not clearly defined so injection was carried out at approximately 25% of the egg length from the posterior pole. As with mosquito and *Drosophila* eggs, significant variation was found in the injection volumes and pressures required for individual *Lu. longipalpis* eggs. This was expected given the asynchronous oviposition of sand fly eggs at varying stages of melanisation.

A total of 1815 eggs were injected with an average of ~300 eggs injected per day. As *Lu. longipalpis* larvae have previously been shown to have variable larval hatching times ^31^, with an average of around 16 days ^34^, a more optimal measure of survivability post-injection was the number of surviving fertile adult G0 females that resulted from the cumulative set of injection experiments. A total of 6 fertile females were produced, which is low compared to the 39 fertile G0 females generated from the injection of 2541 *Ae. aegypti* eggs with the *w*Mel strain of *Wolbachia* ^15^. This low number of fertile adult females is likely a result of the combination of both the egg injection procedure but also the variability of survival rates observed in sandfly colonies due to factors including environmental conditions (temperature and humidity) and parasites and pathogens such as fungi ^31^. PCR analysis revealed *Wolbachia* infections in 3/6 fertile G0 sand fly females. Although screening of G1 progeny from infected females revealed maternal transmission in 2 lines, qPCR cycle threshold values (>32 cycles) indicated low levels present. Selection based on infection status was continued for generation 3 and 4, but no *Wolbachia* infections were detected in the 5 ^th^ generation post-injection.

## Discussion

Insect egg microinjection techniques are dependent on the size and physiology of eggs and additional factors that influence the success rate of experiments, such as preventing excessive egg desiccation, the injection volume and pressure and the use of a buffer to obtain the optimal pH. The developmental stage of the embryo is also critical given eggs that are too young will burst upon injection but fully melanised eggs have a hardened chorion that prevents needle penetration. Ultimately an egg microinjection protocol requires 1) the ability to obtain significant numbers of preblastoderm insect eggs within a short period and 2) a method to rapidly inject eggs with survival of fertile G0 females. The protocol developed here has overcome the first hurdle for egg microinjection in which sufficiently young *Lu. longipalpis* eggs can be harvested for microinjection. Our modified protocol, adapted from a combination of both *Drosophila* and mosquito egg injection techniques, has gravid sandfly females contained within small chambers in close contact with an oviposition substrate. Our results show that successful oviposition occurs despite the absence of a high level of decaying organic matter supporting the previous theory that bacterial volatiles are likely acting as cues for sandfly oviposition ^35^ rather than being directly required on the oviposition surface.

Using *Wolbachia* as a ‘marker’ for successful injection, we were able to generate transient *Wolbachia* infections using our egg injection protocol although on this occasion it was not possible to successfully generate a stably infected line. Despite our optimised protocol producing early generation *Wolbachia* infections, there are several aspects of sand fly biology that are limiting factors for embryonic injection experiments. Firstly, the observation that gravid *Lu. longipalpis* can oviposit fully melanised eggs (and beyond 3 days post-bloodfeed this can be the majority of eggs) would reduce the efficiency of injection due to the necessity for sorting and exclusion of fully melanised eggs. Secondly, the rapid mortality of females shortly after exposure to oviposition substrates and oviposition itself, suggests there is a low probability of obtaining multiple egg batches from any given female. This would reduce the generation of progeny obtained from isofemales in the event no eggs were laid during the first gonotrophic cycle. A recent study looking at multiple gonotrophic cycles in *Lu. longipalpis* demonstrated that oviposition is an essential factor for the success of multiple feeds and outlines a protocol for obtaining sufficient numbers of sandfly females fed on a second blood meal ^36^. Interestingly, the mortality of blood-fed females increased after the second blood meal compared to sugar-fed females suggesting blood feeding results in greater mortality. Finally, the long and asynchronous development of sand fly larvae has implications for the successful mating and bloodfeeding of isofemales. However, it should be possible to overcome these difficulties with a sustained effort to inject large numbers of eggs and the ability to maintain a sand fly colony at high densities.

These preliminary trials to develop an egg microinjection protocol using *Wolbachia* as a ‘marker’ for successful injection resulted in the detection of the *w*Mel strain in G1-G4 generations indicating infection of the ovaries and maternal transmission between generations. The injection of a larger number of sand fly eggs may lead to the successful establishment of transinfected *Wolbachia* lines as has been the case for mosquito eggs ^15, 16, 23, 24^. Resident *Wolbachia* strains are found in some species of sand flies in both field-caught and laboratory colonies ^37– 39^ indicating stable infections could be achievable. *Wolbachia* strains in *Phlebotomus* sand fly colonies have been shown to induce both the reproductive phenotype cytoplasmic incompatibility ^40^ and maternal transmission ^38^, allowing for the invasion of *Wolbachia* into populations. Resident *Wolbachia* strains in mosquitoes have none or only minimal effects on vector competence (reviewed in 41) but transinfection of *Wolbachia* strains from *D. melanogaster* that grow to high densities in mosquito tissues that influence pathogen transmission (e.g. salivary glands) have the greatest inhibitory effects ^15– 17, 42^. Would a high-density strain of *Wolbachia* inhibit *Leishmania* parasites in sand flies? This could only be confirmed through successful generation of a stable line using an efficient egg microinjection protocol as described here given that recent comparative experiments in *Ae. aegypti* mosquitoes have shown that the magnitude of arboviral inhibition is significantly lower in mosquitoes transiently infected with *Wolbachia* using intrathoracic injection into adults ^43^. *Wolbachia* strains have been found to inhibit parasite development in mosquitoes, conferring resistance to *Plasmodium falciparum* malaria infection in *Anopheles stephensi* mosquitoes ^44, 45^ and inhibiting filarial nematode parasite development in *Ae. aegypti* ^46^. The tissue tropism of introduced *Wolbachia* strains in sand flies would be crucial to determine if *Leishmania* parasite development would be inhibited within sand flies. As reviewed in 47, *Leishmania* development is confined to the digestive tract with the production of filamentous proteophosphoglycan in the anterior midgut which creates a gel-like plug. Attachment to the stomodeal valve results in damage to the chitin lining and results in reflux of *Leishmania* parasites from the midgut. Therefore, high density *Wolbachia* infections in the sand fly midgut, as occurs for *Drosophila Wolbachia* strains in *Ae. aegypti* mosquitoes ^15^, would be predicted to result in parasite inhibition.

The ability to inject preblastoderm eggs also provides the possibility of genetic transformation of sand fly species. The widespread success of site-specific nucleases such as transcription activator-like effector nucleases (TALENs) and clustered regularly interspaced short palindromic repeats CRISPR-Cas9 in model organisms such as *D. melanogaster* ^48^ has resulted in research into using reprogrammable gene drive systems based on these nucleases spreading beneficial phenotypes in wild insect populations. This genetic engineering using CRISPR-Cas9 has been used to target all major genera of mosquitoes that transmit human diseases. For example, CRISPR-Cas9 based editing has now been used for the principle vector of dengue and Zika viruses, *Ae. aegypti* ^49^ and has been shown to have the ability to convert female mosquitoes into harmless (non-biting) males ^50^. CRISPR-Cas9 has also been used to explore the potential for the use of transgene drive systems in malaria mosquito vectors. The ability to generate sterile female *Anopheles gambiae* mosquitoes with high transmission rates (>90%) to progeny ^51^ could play a role in modifying wild mosquito populations. In conclusion, this study details an optimised methodology to manipulate bloodfed sand flies to obtain large numbers of *Lu. longipalpis* eggs that are suitable for egg microinjection. Using this method, we showed successful microinjection using *Wolbachia* as a ‘marker’ in the first four generations post infection and provide evidence that that this endosymbiotic bacteria can replicate and be maternally transmitted in *Lu. longipalpis*. However, low survival rates from injection combined with the biology of sandflies does make both stable *Wolbachia* transinfected lines and genetic transformation a significant challenge. Despite these obstacles, this method offers a platform to assess the potential of *Wolbachia* as a novel leishmaniasis biocontrol agent and could also assist in the genetic manipulation of this important vector of leishmaniasis.

## Data availability

Raw data is available at Open Science Framework: http://doi.org/10.17605/OSF.IO/S7CZP ^52^

Data are available under the terms of the Creative Commons Zero “No rights reserved” data waiver (CC0 1.0 Public domain dedication).
