# Supplementary material for: Establishment of a method for Lutzomyia longipalpis sand fly egg microinjection: The first step towards potential novel control strategies for leishmaniasis
Source: Wellcome Open Res. 2018 Aug 15;3:55. Originally published 2018 May 9. [Version 2] doi: 10.12688/wellcomeopenres.14555.2 (PMC6107984; doi:10.12688/wellcomeopenres.14555.2)
Supplement: Peer review history

# Peer review: Referee response for version 2

**DOI:** 10.21956/wellcomeopenres.16029.r33697

**Published:** 2018-08-22

**Authors:** Marcelo Ramalho-Ortigao

**Affiliations:** 1 F. Edward Hebert School of Medicine, Department of Preventive Medicine and Biostatistics, Uniformed Services University of the Health Sciences (USUHS), Bethesda, MD, USA

I am happy with the changes made by the authors. However,  I noticed two very small “typos” and details that that would require correction:

  

1. In the section “ ***Wolbachia* purification and egg injection**”, on the first sentence of the paragraph it should read **“…**by gently crushing ~10 pairs of dissected…”. There is a “x” with number 10 (this was pointed out on the first review, and might have been overlooked).
2. In the text, both **sand fly/sand flies** and **sandfly/sandflies **are used. One format should be used.
3. In the revised version of the manuscript, I still see “principle” when referring to Lu. longipalpis in the Abstract. It should be **principal.**
4. The word p **rinciple** also appears in the Discussion when referring to Aedes aegypti: “For example, CRISPR-Cas9 based editing has now been used for the principle vector of dengue and Zika viruses, *Ae. aegypti*”. Here, too, should be either **principal** or **major** (preferably the latter).

I have read this submission. I believe that I have an appropriate level of expertise to confirm that it is of an acceptable scientific standard.

# Peer review: Referee response for version 1

**DOI:** 10.21956/wellcomeopenres.15842.r33395

**Published:** 2018-07-13

**Authors:** Marcelo Ramalho-Ortigao

**Affiliations:** 1 F. Edward Hebert School of Medicine, Department of Preventive Medicine and Biostatistics, Uniformed Services University of the Health Sciences (USUHS), Bethesda, MD, USA

Review of manuscript “ Establishment of a method for *Lutzomyia longipalpis* sand fly embryo microinjection: The first step towards potential novel control strategies for leishmaniasis”, by Jeffries CL, Rogers ME and Walker T

 The study by Jeffries et al described a methodology for sorting and microinjecting sand fly eggs. The authors use *Wolbachia* wMel as a tracer as this bacterium is not found in natural sand fly populations. Though the authors manage to inject a large number of eggs, the overall rate of survival, estimated from the number of fertile females obtained (six), is very low. The study is a step in the right direction, providing a road map to investigators interested in sand fly biology to adopt techniques currently shown to be successful for mosquitoes and mosquito-borne pathogens, and I congratulate the authors on their efforts. However, there are a number of statements made throughout the manuscript which are misleading or inaccurate, and need to be corrected. Moreover, the results described to some extent “miss the mark” with regards to a number of critical details.

 Comments

**Major issues**

  

- There is a back and forth between melanized eggs and embryos when in actuality the authors should be referring to melanized eggs or chorion, not embryos. “Melanized embryo” is not used correctly and sand fly embryo melanization process was not observed by the authors. Here, egg shell darkening was used as reference of suitability of embryo for microinjection. Whereas melanization of the chorion hardens the egg making it difficult to pierce through the egg shell during microinjection and likely leading to additional damage, such as desiccation of the embryo following piercing or injection, melanization of the embryo itself may occur as an innate immune response.
- The authors use “degree of chorion melanization” as an index to score injectable eggs but this was never paired with any observation of embryo development.  
- The rate of survival following injection of the eggs is still extremely low, with a reported 6 fertile females after 1815 eggs injected (0.33% survival). Any idea on the hatching rate [it was not indicated]? It appears as though the authors have compared their 6 fertile sand fly females obtained here with the results obtained by Walker et al. 2011 with *Aedes aegypti* transfected with wMel. In that study, there were a total of 69 G0 females, 39 of which were fertile. However, only 13 were wMel infected. As indicated on page 8, only 6 fertile females were obtained. The ratio of fertile females in the Walker et al study is 1.5%, with a survival rate (total number of females) of 2.71%. The assumption that rate of survival of fertile females is comparable (5 fold difference) might be a bit misleading. 
- As authors mentioned, melanized eggs are seldom observed before oviposition in *L. longipalpis*. This phenomenon has not been well understood. Although, a decreasing percentage of suitable eggs for injection obtained longer after blood meal might suggest chorion melanization process is loosely related to oviposition in this sand fly species. Hence, it represents a challenge for the proposed procedure for egg microinjection. 
- Authors should be able to present results from screening of wMel strain of *Wolbachia* in G1-G5 for a better understanding of this transient colonization of the bacteria in the isofemale lines. 
- Were flies assessed for survival following oviposition while provided with sugar solution during the experiments (i.e., during all the days they were in the ovipots)? This is in reference to results presented in Figure 4. It is not clear from the text whether flies were or were not offered sugar solution. 
- Figure 3 legend: (b) the total number of eggs collected, not embryos. 
- Figure 2: picture of larvae referred to as “larvae hatching on oviposition substrate” shows what appears to be a L3 (with 2 sets of caudal setae). Either clarify this in the figure legend or replace it with and actual figure of a “hatching larva”. As indicated elsewhere in this review, the melanization takes place in the chorion of the egg and not the embryo. This should be corrected for this figure. 
- In the results, the authors indicate that “…gravid sand flies can retain viable mature embryos [in actuality it should be eggs] until an appropriate substrate is available…” Though I agree with this statement, what is lacking here are details of “substrate”. Part of this study was focused on comparing the effects of 2% agarose vs. plaster on egg laying and it appears as though the focus of this statement is on such difference. However, it has been demonstrated that bacteria present in the substrate (typically decaying organic matter) are key for oviposition by sand flies, and this is not mentioned by the authors. In my view, the effect of bacteria on sand fly oviposition also needs to be discussed if trying to interpret the effects of “appropriate substrate”. 

**Other issues needing correction**

**Abstract**

 “…L. longiplais is the principal vector…”

 “…handling of small sand fly eggs…”

 It needs to indicate that the transfection with Wolbachia was transient (per the results until the 5 ^th^ generation).

**Introduction**

  

- Typically, the spectrum of diseases is referred to as leishmaniases (with an “e”).

 

- The sentence explaining aspects of the clinical symptoms (or as written “syndrome”) should be re-written for accuracy. Human migration is related to transmission of disease and not “extrinsic” factors associated with the clinical symptoms.

 

- For the statement of clinical symptoms: “The clinical syndrome exhibited is influenced by the species of the infecting parasites, the genetic background of the host and associated immunity.”

 

- On paragraph 3 of the Intro: In endemic areas where dogs are domestic reservoirs of **visceral** leishmaniasis (not cutaneous!) 
- It seems to me that the paragraph starting with “Research into novel non-insecticide based control strategies…” should be part of the Discussion.

 

**Methods**

L. longipalpis rearing

 Jacobina is not a state in Brazil. The state is actually Bahia; Jacobina is a city. The coordinated for Jacobina, though not necessarily where the flies were originally collected is 11° 10′ 51″ S, 40° 31′ 4″ W.

 Under laboratory conditions, hatching of *Lutzomyia longipalpis* varies widely and is dependent on temperature and relative humidity. However, this is one of the most malleable species for colonization. Perhaps the authors want to add comments as to whether rearing methodology and subsequent mating procedure may have contributed to a low yield of fertile adult G0 females.

Wolbachia purification and embryo injection

 There are two references cited in this section, numbers 32 and 15. Following the journal’s style, they should be superscript, perhaps by citing “author et al” (e.g., Klasson et al ^32^; Walket et al ^15^)

 “…by crushing x10 pairs…”. Did you mean crushing ~10 pairs? (“~” as in approximately?)

Isofemale line selection

 “…45 degree angle perpendicular to the plaster base…” If lines are perpendicular they are by definition at a 90° degree (right) angle.

 In the section where the PCR details are described, please be consistent in the use of µl: there is µl, µL. Also, separate µl and the number digit, as in 1 µl, not 1µl.

**Discussion**

 Though the techniques described in this study are a step in the right direction, to “predict” that using Wolbachia will lead to inhibition of Leishmania growth in sand flies based on details of its biology seems a bit of a stretch (although I would like to agree with the authors!). In addition, the authors go on to suggest that CRISPR-Cas9 will be the next natural approach to be tested and that the “optimized” methodology described will allow for the manipulation of large numbers of Lu. longipalpis embryos. The authors are correct with regards to CRISPR, as a handful of labs are already applying this approach to their study of sand flies. However, the success rate is still dismal to say the least. Until the survival rate and the rate of fertile females are higher that what was reported here, it is difficult to see this moving rapidly to be applied in any control, strategies for sand fly-transmitted diseases. In that regard, I do not think the picture is as rosy.

I have read this submission. I believe that I have an appropriate level of expertise to confirm that it is of an acceptable scientific standard, however I have significant reservations, as outlined above.

# Response

**Published:** 2018-08-07

**Authors:** Thomas Walker

**Affiliations:** London School of Hygiene & Tropical Medicine, UK

Many thanks Marcelo for your insightful review.  We have addressed your comments as follows  (in bold): 

There is a back and forth between melanized eggs and embryos when in actuality the authors should be referring to melanized eggs or chorion, not embryos.

**We have gone through the manuscript and corrected our terminology on eggs vs embryos as we agree that these are distinct terms.**

The authors use “degree of chorion melanization” as an index to score injectable eggs but this was never paired with any observation of embryo development.

**We did not do this as the use of melanisation to determine ‘injectability’ is commonly used for mosquito injection methodology and provides a ‘usable’ guide as to when to inject embryos independent of embryo development (please see Jasinskiene, N., Juhn, J. & James, A. A. Microinjection of A. aegypti embryos to Obtain Transgenic Mosquitoes. *J. Vis. Exp.* (2007).  To determine embryo development for any individual in the time frame required to inject would not be feasible.**

The rate of survival following injection of the eggs is still extremely low, with a reported 6 fertile females after 1815 eggs injected (0.33% survival). Any idea on the hatching rate [it was not indicated]? It appears as though the authors have compared their 6 fertile sand fly females obtained here with the results obtained by Walker et al. 2011 with *Aedes aegypti* transfected with wMel. In that study, there were a total of 69 G0 females, 39 of which were fertile. However, only 13 were wMel infected. As indicated on page 8, only 6 fertile females were obtained. The ratio of fertile females in the Walker et al study is 1.5%, with a survival rate (total number of females) of 2.71%. The assumption that rate of survival of fertile females is comparable (5 fold difference) might be a bit misleading.

**We agree and have modified our text to reflect this slightly misleading comparison to Walker et al. study.  “A total of 6 fertile females were produced, which is low compared to the 39 fertile females generated from the injection of 2541 *Ae. aegypti*eggs with the *w*Mel strain of *Wolbachia* ^15^.” We also have addressed the idea that rearing methodology can contribute to low yield of fertile adult G0 females “The low number of fertile adult females is likely a result of the combination of both the egg injection procedure but also the variability of survival rates observed in sandfly colonies due to factors including environmental conditions (temperature and humidity) and parasites and pathogens such as fungi ^31^.”**

As authors mentioned, melanized eggs are seldom observed before oviposition in *L. longipalpis*. This phenomenon has not been well understood. Although, a decreasing percentage of suitable eggs for injection obtained longer after blood meal might suggest chorion melanization process is loosely related to oviposition in this sand fly species. Hence, it represents a challenge for the proposed procedure for egg microinjection. 

**We agree and we do mention this in the discussion with the following sentences “Firstly, the observation that gravid *Lu.* *longipalpis *can oviposit fully melanised eggs (and beyond 3 days post-bloodfeed this can be the majority of eggs) would reduce the efficiency of injection due to the necessity for sorting and exclusion of fully melanised eggs.  Secondly, the rapid mortality of females shortly after exposure to oviposition substrates and oviposition itself, suggests there is a low probability of obtaining multiple egg batches from any given female.”**

Authors should be able to present results from screening of wMel strain of *Wolbachia* in G1-G5 for a better understanding of this transient colonization of the bacteria in the isofemale lines.

**As the manuscript is a ‘methods’ paper that is presenting *Wolbachia* as a ‘marker’ for successful germline infection (rather than establishing a stable line), this data was omitted as it has limited relevance to the injection protocol.  The asynchronous development of sandfly immature stages in combination with published studies establishing *Wolbachia*-infected mosquito lines showing huge variation in maternal transmission rates in early generations (Walker et al. Nature 2011, Joubert et al Plos Pathogens 2016) suggests this data is not very informative.**

Were flies assessed for survival following oviposition while provided with sugar solution during the experiments (i.e., during all the days they were in the ovipots)? This is in reference to results presented in Figure 4. It is not clear from the text whether flies were or were not offered sugar solution. 

**During the short oviposition collections (45 minutes) the flies were not offered a sugar solution but were maintained on sugar before and after in cages. In our manuscript we have “ Selected females were maintained within oviposition chambers overnight by the addition of sugar-soaked cotton wool to the mesh, and with replacement of oviposition substrate plates with empty falcon tube lids if it was desirable to prevent additional oviposition overnight” We also explain that ‘high adult female mortality in colony *Lu. longipalpis *shortly after egg-laying was also observed’ suggesting the absence of sugar for only the ‘oviposition’ period is unlikely to be the cause of this mortality.**

Figure 3 legend: (b) the total number of eggs collected, not embryos

**We have changed the terminology here.**

picture of larvae referred to as “larvae hatching on oviposition substrate” shows what appears to be a L3 (with 2 sets of caudal setae). Either clarify this in the figure legend or replace it with and actual figure of a “hatching larva”. As indicated elsewhere in this review, the melanization takes place in the chorion of the egg and not the embryo. This should be corrected for this figure

**This larvae was from the hatching experiment and was erroneously included in this figure. We have removed this image and modified figure 2 which does actually have evidence of a larvae hatching in the revised figure in panel F (this was also in the original figure).  We have also adjusted our terminology of egg rather than embryo for melanization.**

In the results, the authors indicate that “…gravid sand flies can retain viable mature embryos [in actuality it should be eggs] until an appropriate substrate is available…” Though I agree with this statement, what is lacking here are details of “substrate”. Part of this study was focused on comparing the effects of 2% agarose vs. plaster on egg laying and it appears as though the focus of this statement is on such difference. However, it has been demonstrated that bacteria present in the substrate (typically decaying organic matter) are key for oviposition by sand flies, and this is not mentioned by the authors. In my view, the effect of bacteria on sand fly oviposition also needs to be discussed if trying to interpret the effects of “appropriate substrate”. 

**Our oviposition substrates were freshly made and kept at 4C so there was unlikely to be large amounts of bacteria present on the substrates.  We fully agree that decaying organic matter is important for oviposition so it’s possible that our method can circumvent this need through ‘forced oviposition’ rather than attracting sandflies to oviposit through bacterial volaties.  We have added a sentence to the manuscript to address this point.**

“…L. longiplais is the principal vector…”

**Changed in ms**

“…handling of small sand fly eggs…”

**Changed in ms**

It needs to indicate that the transfection with Wolbachia was transient (per the results until the 5 ^th^generation).

**‘Transient’ is used for Wolbachia transinfection work as meaning no maternal transmission (ie. through adult intrathoracic injection).   Here Wolbachia was maternally transmitted through four generations so we feel ‘transient’ does not represent our results but early generation indicates the absence of a stable line. ** 

·       Typically, the spectrum of diseases is referred to as leishmaniases (with an “e”).

**Changed in ms**

·       The sentence explaining aspects of the clinical symptoms (or as written “syndrome”) should be re-written for accuracy. Human migration is related to transmission of disease and not “extrinsic” factors associated with the clinical symptoms.

· **We have re-written this sentence**

For the statement of clinical symptoms: “The clinical syndrome exhibited is influenced by the species of the infecting parasites, the genetic background of the host and associated immunity

· **We have re-written this sentence**

·       On paragraph 3 of the Intro: In endemic areas where dogs are domestic reservoirs of visceralleishmaniasis (not cutaneous!)

· **This was a mistake and we have changed this!**

****

·       It seems to me that the paragraph starting with “Research into novel non-insecticide based control strategies…” should be part of the Discussion.

**We respectfully disagree as this introduces the idea of needing novel control strategies in sandflies and the requirement for egg injection for various strategies currently being used for mosquito control**

Jacobina is not a state in Brazil. The state is actually Bahia; Jacobina is a city. The coordinated for Jacobina, though not necessarily where the flies were originally collected is 11° 10′ 51″ S, 40° 31′ 4″ W.

**We have modified this in the ms**

Under laboratory conditions, hatching of *Lutzomyia longipalpis* varies widely and is dependent on temperature and relative humidity. However, this is one of the most malleable species for colonization. Perhaps the authors want to add comments as to whether rearing methodology and subsequent mating procedure may have contributed to a low yield of fertile adult G0 females.

**We feel this is a good point to raise and have addressed this in the manuscript**

There are two references cited in this section, numbers 32 and 15. Following the journal’s style, they should be superscript, perhaps by citing “author et al” (e.g., Klasson et al ^32^; Walket et al ^15^)

**We have modified this in the ms**

“…by crushing x10 pairs…”. Did you mean crushing ~10 pairs? (“~” as in approximately?)

**We have clarified this – 10 pairs of ovaries**

“…45 degree angle perpendicular to the plaster base…” If lines are perpendicular they are by definition at a 90° degree (right) angle.

**We have removed ‘perpendicular’ to address this mistake**

In the section where the PCR details are described, please be consistent in the use of µl: there is µl, µL. Also, separate µl and the number digit, as in 1 µl, not 1µl.

**We have corrected this in the ms**

Though the techniques described in this study are a step in the right direction, to “predict” that using Wolbachia will lead to inhibition of Leishmania growth in sand flies based on details of its biology seems a bit of a stretch (although I would like to agree with the authors!). In addition, the authors go on to suggest that CRISPR-Cas9 will be the next natural approach to be tested and that the “optimized” methodology described will allow for the manipulation of large numbers of Lu. longipalpis embryos. The authors are correct with regards to CRISPR, as a handful of labs are already applying this approach to their study of sand flies. However, the success rate is still dismal to say the least. Until the survival rate and the rate of fertile females are higher that what was reported here, it is difficult to see this moving rapidly to be applied in any control, strategies for sand fly-transmitted diseases. In that regard, I do not think the picture is as rosy.

**We agree and have sentences in the discussion such as ‘The injection of a larger number of sand fly embryos may lead to the successful establishment of transinfected *Wolbachia* lines as has been the case for mosquito embryos’ where ‘’may’’ is used to provide balance. We also have ‘Would a high-density strain of *Wolbachia* inhibit *Leishmania* parasites in sand flies? This could only be confirmed through successful generation of a stable line…’  and ‘The tissue tropism of introduced *Wolbachia* strains in sand flies would be crucial to determine if *Leishmania* parasite development would be inhibited within sand flies’ so feel we do have some balance in our discussion. We are unable to comment on the success of ongoing CRISPR studies that are currently unpublished but agree that many technical hurdles may still have to be overcome and have added a further sentence to the end of the discussion.**

# Peer review: Referee response for version 1

**DOI:** 10.21956/wellcomeopenres.15842.r33300

**Published:** 2018-06-22

**Authors:** Fernando Ariel Genta

**Affiliations:** 1 Laboratory of Insect Physiology and Biochemistry, Oswaldo Cruz Institute - Oswaldo Cruz Foundation (IOC-FIOCRUZ), Rio de Janeiro, Brazil

This article by Jeffries *et al.* describes a new methodology for obtaining embryos of the phlebotomine sand fly *Lutzomyia longipalpis*, an important vector of Visceral Leishmaniasis in South America. The method described allowed the authors to do injections in these embryos at a very large scale. This method will be useful for genetic manipulations of this insect, and it was applied to inoculation of Wolbachia in a strain not previously associated with this bacterium species. Despite the fact that the authors failed in obtaining a stable Wolbachia-sand fly association, the technical achievement described is very relevant to the sand fly community. I have only very few remarks to do about this manuscript, listed below:

 1. Abstract line 9 - "principal" instead of "principle"

 2. Results page 6, sentence "These initial tests..." I understand that they are initial tests, but I missed a more detailed description and comparison of the data. How much better was the chosen condition in comparison to the others? How many eggs were laid in the three media tested? How strong was the effect of light and sand fly number? These considerations would be important for developers and to have a better understanding of the sand fly biology involved.

 3. Figure 3. The legend of the figure does not describe all charts in part b. Maybe it is just a problem in the colors described, they do not match all the pizza slices in the figure.

 4. Discussion page 9. Sentence "Secondly, the rapid mortality..." We have just published a report showing second blood feeding and oviposition in sand flies ( *Lutzomyia longipalpis* of the very same strain used in this article) in large-scale experiments with the same efficiency when compared to the first blood meal/oviposition cycle ^1^. I think our finding might be useful for the authors in the future and it would be interesting to mention them in this context.

 I would like to congratulate the authors for this manuscript, it is a quite interesting and relevant work.

I have read this submission. I believe that I have an appropriate level of expertise to confirm that it is of an acceptable scientific standard.

## References

[1] MoraesCSAguiar-MartinsKCostaSGBatesPADillonRJGentaFA: Second Blood Meal by Female Lutzomyia longipalpis: Enhancement by Oviposition and Its Effects on Digestion, Longevity, and Leishmania Infection. *Biomed Res Int*.2018;2018: 10.1155/2018/2472508 2472508 10.1155/2018/2472508 29770328PMC5889884

# Response

**Published:** 2018-08-07

**Authors:** Thomas Walker

**Affiliations:** London School of Hygiene & Tropical Medicine, UK

Many thanks Fernando for your review and we have addressed your comments as follows (in bold): 

 Abstract line 9 - "principal" instead of "principle"

**We have changed this in the manuscript**

 Results page 6, sentence "These initial tests..." I understand that they are initial tests, but I missed a more detailed description and comparison of the data. How much better was the chosen condition in comparison to the others? How many eggs were laid in the three media tested? How strong was the effect of light and sand fly number? These considerations would be important for developers and to have a better understanding of the sand fly biology involved.

**This was indeed more observational during initial tests to indicate the suitability of using agar as a substrate for oviposition (plaster of paris used for colony maintenance).  As we found that sandflies laid a significant proportion of melanised eggs during the initial trials, we went on to determine the proportion of ‘injectable’ eggs on days 3-5 post-bloodfeed (Figure 3, raw data file) as that would have a greater impact on the ability to undertake this work rather than overall numbers of eggs laid.**

 Figure 3. The legend of the figure does not describe all charts in part b. Maybe it is just a problem in the colors described, they do not match all the pizza slices in the figure.

**We agree and have modified the figure legend to clarify the difference between egg colour from melanisation and colours on the charts! ** 

 Discussion page 9. Sentence "Secondly, the rapid mortality..." We have just published a report showing second blood feeding and oviposition in sand flies ( *Lutzomyia longipalpis* of the very same strain used in this article) in large-scale experiments with the same efficiency when compared to the first blood meal/oviposition cycle ^1^. I think our finding might be useful for the authors in the future and it would be interesting to mention them in this context.

**We have now included this result and reference in our discussion.**

****
